# *Entamoeba histolytica*-secreted cysteine proteases induce IL-8 production in human mast cells via a PAR2-independent mechanism

**DOI:** 10.1051/parasite/2014001

**Published:** 2014-02-07

**Authors:** Young Ah Lee, Young Hee Nam, Arim Min, Kyeong Ah Kim, Tomoyoshi Nozaki, Yumiko Saito-Nakano, David Mirelman, Myeong Heon Shin

**Affiliations:** 1 Department of Environmental Medical Biology, Institute of Tropical Medicine, and Brain Korea 21 Project for Medical Science, Yonsei University College of Medicine Seoul 120-752 Korea; 2 Department of Parasitology, National Institute of Infectious Diseases, 1-23-1 Toyama Shinjuku-ku, Tokyo Japan; 3 Department of Biological Chemistry, Weizmann Institute of Science Rehovot 76100 Israel

**Keywords:** *Entamoeba histolytica*, Cysteine protease, Mast cell, IL-8, Protease-activated receptor 2 (PAR2)

## Abstract

*Entamoeba histolytica* is an extracellular tissue parasite causing colitis and occasional liver abscess in humans. *E. histolytica*-derived secretory products (SPs) contain large amounts of cysteine proteases (CPs), one of the important amoebic virulence factors. Although tissue-residing mast cells play an important role in the mucosal inflammatory response to this pathogen, it is not known whether the SPs induce mast cell activation. In this study, when human mast cells (HMC-1 cells) were stimulated with SPs collected from pathogenic wild-type amoebae, interleukin IL-8 mRNA expression and production were significantly increased compared with cells incubated with medium alone. Inhibition of CP activity in the SPs with heat or the CP inhibitor E64 resulted in significant reduction of IL-8 production. Moreover, SPs obtained from inhibitors of cysteine protease (ICP)-overexpressing amoebae with low CP activity showed weaker stimulatory effects on IL-8 production than the wild-type control. Preincubation of HMC-1 cells with antibodies to human protease-activated receptor 2 (PAR2) did not affect the SP-induced IL-8 production. These results suggest that cysteine proteases in *E. histolytica*-derived secretory products stimulate mast cells to produce IL-8 via a PAR2-independent mechanism, which contributes to IL-8-mediated tissue inflammatory responses during the early phase of human amoebiasis.

## Abstract (fr)

*Entamoeba histolytica* est un parasite extracellulaire des tissus provoquant des colites et occasionnellement des abcès du foie chez l’homme. Les produits de sécrétion dérivés d’*E. histolytica* (SPs) contiennent de grandes quantités de cystéine-protéases (CPs), l’un des principaux facteurs de virulence amibiens. Bien que les mastocytes tissulaires jouent un rôle important dans la réponse inflammatoire de la muqueuse à ce pathogène, on ne sait pas si les SPs induisent l’activation des mastocytes. Dans cette étude, lorsque des mastocytes humains (cellules HMC-1) ont été stimulés avec des SPs recueillis à partir d’amibes pathogènes de type sauvage, l’expression et la production de l’interleukine IL-8 ont été significativement augmentées par rapport à des cellules incubées avec du milieu seul. L’inhibition de l’activité des CPs dans les SPs avec la chaleur ou avec E64, un inhibiteur de CP, a entraîné une réduction significative de la production d’IL-8. En outre, les SPs obtenus à partir d’amibes surexprimant l’inhibiteur de protéases à cystéine (ICP) à faible activité de CP ont montré des effets stimulants plus faibles sur la production d’IL-8 que le contrôle de type sauvage. La pré-incubation des cellules HMC-1 avec des anticorps contre le récepteur 2 activé par la protéase humaine (PAR2) n’a pas affecté la production d’IL-8 induite par SPs. Ces résultats suggèrent que les cystéine-protéases des produits de sécrétion dérivés d’*E. histolytica* stimulent les mastocytes pour produire de l’IL-8 par l’intermédiaire d’un mécanisme indépendant de PAR2, ce qui contribue à la réponse inflammatoire tissulaire médiée par IL-8 au cours de la phase précoce de l’amibiase humaine.

## Introduction

The enteric protozoan parasite *Entamoeba histolytica* Schaudinn, 1903 [25] is the causative agent of human amoebiasis. *E. histolytica* trophozoites bind colonic mucin via amoebic Gal-lectin and subsequently cause mucin degradation and colonic epithelial cell death through apoptosis or necrosis [19, 20]. *E. histolytica* releases various proteases into the extracellular milieu. In particular, cysteine proteases (CPs) are abundant in *E. histolytica*-derived secretory products (SPs). CPs are important for host mucin and extracellular matrix (ECM) degradation [11], cytopathic effects on the host cell, and activation of IL-1 β or IL-18.

*E. histolytica*-induced colon cell death promotes IL-8-mediated acute tissue inflammation at the site of infection [26]. IL-8 is a potent chemoattractant and activator of neutrophils and can cause non-specific tissue damage after activation [9]. Although neutrophils are the primary target cells of IL-8, IL-8 has other effects on various kinds of cells such as endothelial cells, other granulocytes, macrophages, and mast cells. The infiltration of immune cells including neutrophils, macrophages, and mast cells at the mucosal surface was observed during *E. histolytica* intestinal amoebiasis, suggesting that these cells might be important in host defense against this parasite [13]. Moreover, an increase in degranulation and disruption of mast cells was reported in *E. histolytica*–infected mice [16], suggesting that mast cells play a role in *E. histolytica*–induced tissue inflammation at the inflamed site. However, the precise role of amoebic CPs in mast cell activation is poorly understood.

Mast cells contribute to the innate and adaptive host defense mechanisms through the release of an arsenal of inflammatory mediators upon activation by various stimuli [3, 12]. Tissue-residing mast cells are major players in the mucosal inflammatory response to various bacterial and parasitic infections [6]. Activated mast cells release various proinflammatory mediators including histamine, IL-6, IL-8, IL-13, prostaglandin D2, leukotriene C4 (LTC4), and tumor necrosis factor-α (TNF-α) in response to various stimuli [14]. The essential role of mast cells in the host control of infection has been shown in animal models infected with various bacterial and parasitic pathogens [2, 4, 28]. However, little is known about the effect of amoebic SPs on IL-8 secretion in mast cells.

In this study, mast cells were stimulated with *E. histolytica-*derived SPs from *E. histolytica* wild-type or mutant strains to ascertain if SPs directly induce IL-8 production. The results of this work show that amoebic CPs participate in SP-induced IL-8 production in HMC-1 cells.

## Materials and methods

### Reagents

Unless stated otherwise, all other chemicals were purchased from Sigma-Aldrich (Saint Louis, MO, USA). Mouse monoclonal antibody (Ab) against human protease-activated receptor 2 (PAR2) (SAM 11) and normal mouse IgG_2a_ were purchased from Santa Cruz Biotechnology (Santa Cruz, CA, USA). Fluorescent isothiocyanate (FITC)-labeled annexin V, PE-conjugated anti-human CD63, and PE-conjugated anti-mouse IgG_1_κ were purchased from BD Pharmingen (San Diego, CA, USA). Dichlorodihydrofluorescein diacetate (H_2_DCFDA) was purchased from Molecular Probes (Eugene, OR, USA).

### Cultivation of human HMC-1 cells

The HMC-1 human mast cell line was grown in Iscove’s Modified Dulbecco’s medium (IMDM) (Invitrogen) containing 10% (v/v) heat-inactivated fetal bovine serum (FBS) and 0.5% penicillin-streptomycin at 37 °C in a humidified 5% CO_2_ atmosphere. HMC-1 cell viability, as judged by trypan blue exclusion testing, was 99%.

### Culture of bone marrow-derived murine mast cells (BMMCs) and ethics

BMMCs from BALB/c mice (Orient Bio, Seoul, Korea) were obtained by culturing mouse femoral bone marrow cells in RPMI containing 10% (v/v) heat-inactivated fetal bovine serum, 0.5% penicillin-streptomycin, 50 μM β-mercaptoethanol, and 20 ng/mL IL-3 (PeproTech, Rocky Hill, NJ, USA) for 4 weeks. At that time, the cells were > 98% c-kit^high^ FcεRI^high^, according to flow cytometric analysis using PE anti-mouse FcεRIa (MAR-1) (eBioscience, San Diego, CA, USA) and FITC anti-mouse c-kit/CD117 (2D8) (eBioscience, San Diego, CA, USA). The present study was approved by the Yonsei University Animal Research Ethics Committee (No. 2013-0297).

### Construction of *E. histolytica* cell lines overexpressing a GFP-tagged inhibitor of cysteine protease 1 (ICP1^+/+^)

To create the ICP1-overexpressing *E. histolytica* cell line (ICP1^+/+^), a full-length *E. histolytica ICP1* gene was amplified by PCR from cDNA using sense and antisense oligonucleotides containing appropriate restriction sites (underlined) at the 5′-end: *icp1* 5′-aatcccgggATGTCATTAACTGAAGATAATAACAACACAAC-3′ (*Sma*I); and 5′-gggctcgagTTACTGGACATTAACTTTTAAAGTAAAAG-3′ (*Xho*I). The PCR-amplified DNA fragments were digested and ligated into the same restriction sites of the overexpression vector, pKT-MG. This vector allows for the expression of a gene of interest as a N-terminal fusion with GFP. The plasmids were introduced into *E. histolytica* HM-1:IMSS trophozoites by liposome-mediated transfection as previously described [22], and stable transformants were cultured in medium containing 8 μg/mL G418 (for ICP1^+/+^ and pKT-MG as a vector control).

### Cultivation of *E. histolytica* trophozoites and preparation of secretory products

Trophozoites of the *E. histolytica* virulent HM-1:IMSS strain, the avirulent *E. histolytica* Rahman strain, and the hypo-CP strain ICP1^+/+^ were grown at 37 °C in TYI-S-33 medium as described previously [10]. After cultivation for 48–72 h, logarithmic growth phase trophozoites were harvested by incubation on ice for 10 min, followed by centrifugation at 1000 rpm at 4 °C for 5 min. To collect SPs, trophozoites from the various strains were incubated in Hanks’ balanced salt solution (GIBCO Laboratories, Grand Island, NY, USA) for 2 h at 37 °C at a final concentration of 1 × 10^7^ amoebae per mL. The viability of *Entamoeba* trophozoites after incubation in Hank’s balanced salt solution was 99% as determined by the trypan blue exclusion assay. Protein concentration was measured by the BCA protein assay using bovine serum albumin as a standard.

### Measurement of cell viability and cell death

HMC-1 cell viability and cell death were measured by trypan blue and annexin V/propidium iodide (PI) double staining, respectively. HMC-1 cells (5 × 10^5^ cells/sample) stimulated with SPs were incubated for 2 h at 37 °C in a humidified CO_2_ incubator (5% CO_2_ and 95% air). After incubation, the reaction was stopped by brief centrifugation, and the cells were washed with cold PBS twice and stained with FITC-conjugated annex in V and 1 μg/mL PI. Flow cytometric analysis was performed with a FACScan on at least 10,000 cells from the host cell fraction.

### Measurement of exocytosis in HMC-1 cells or BMMCs

To elucidate the exocytosis of HMC-1 cells or BMMCs induced by the SPs, HMC-1 cells or BMMCs (5 × 10^5^ cells/sample) were incubated with SP (30 or 100 μg/mL) for 2 h, or platelet-activating factor (PAF) (5 μM) for 1 h or phorbol 12-myristate 13-acetate (PMA) for 2 h. For positive control, BMMCs were treated with monoclonal anti-DNP IgE (250 ng/mL) for 4 h, and then the cells were challenged with DNP-HSA (250 ng/mL) for 1 h to trigger mast cell degranulation. After incubation, the cells were washed twice with washing buffer (0.1% sodium azide and 1% FBS in PBS) and stained with PE-conjugated anti-human or anti-mouse CD63. PE-conjugated anti-mouse IgG_1_ or anti-rat IgG_2a_ was used as an isotype control. A flow cytometric analysis was performed using a FACScan on at least 10,000 cells from each sample.

### Quantitative real-time PCR for IL-8 mRNA expression

HMC-1 cells (1 × 10^6^/sample) were incubated with or without SPs for 30 min at 37 °C in a CO_2_ incubator. After incubation, total RNA was obtained from HMC-1 cells (1 × 10^6^/sample) incubated with or without SPs using TRIzol reagent (Invitrogen Corporation, Carlsbad, CA, USA) and was reverse-transcribed using a ProSTAR first-strand RT-PCR kit (Stratagene, La Jolla, CA, USA). Obtained cDNA was amplified using a SYBR® Green PCR Master Mix. The primers used were as follows: human IL-8; 5′-TCT GCA GCT CTG TGT GAA GGT G-3′ and 5′-AAT TTC TGT GTT GGC GCA GTG-3′, human GAPDH; 5′-GAA GGT GAA GGT CGG AGT C-3′ and 5′-GAA GAT GGT GAT GGG ATT TC-3′. IL-8 gene expression was analyzed using the Applied Biosystems 7700 Sequence Detection System (Applied Biosystems, Foster City, CA, USA), according to the manufacturer’s instructions. The relative amount of mRNA for the genes of interest was determined by subtracting the threshold cycle (Ct) values for the gene from the Ct value for the internal control gene Glyceraldehyde-3-phosphate dehydrogenase (GAPDH) (ΔCt). Each measurement of a sample was performed in triplicate. The data represent IL-8 mRNA fold induction.

### IL-8 ELISA

For measurement of IL-8 production, HMC-1 cells (5 × 10^5^/well) were seeded in 24-well tissue culture plates and then directly incubated for the indicated times with or without native or modified SPs for 12 h at 37 °C in a CO_2_ incubator. After incubation, culture supernatants were collected from HMC-1 cells. To evaluate the involvement of PAR2 in SP-induced IL-8 production, HMC-1 cells (5 × 10^5^/well) were preincubated with anti-PAR2 (10 μg/mL) or mouse IgG_2a_ (10 μg/mL) for 30 min. After preincubation, the cells were incubated for the indicated times with or without native SPs for 12 h at 37 °C in a CO_2_ incubator. Then, culture supernatants were collected from HMC-1 cells, and the amount of IL-8 production was measured with a specific human IL-8 screening kit (Thermo Scientific, Waltham, MA, USA) according to the manufacturer’s instructions.

### Measurement of cysteine protease activity

Native SPs obtained from wild-type amoebae were pretreated with or without protease inhibitor (E64 or PMSF) or heat-modified SPs (100 °C for 10 min), and then CP assays were performed. In addition, ICP1^+/+^, vector control, or Rahman-derived native SPs were also measured for CP activity. Briefly, *E. histolytica* trophozoites (4 × 10^5^/well) were incubated in 100 mL Opti-MEM medium (Invitrogen, Carlsbad, CA, USA) supplemented with 137 mM cysteine and 19 mM ascorbic acid, pH 6.8 in a 96-well tissue culture plate at 37 °C for 1 h. After incubation, the culture supernatant for secreted CP activity was collected and remaining trophozoites for intracellular CP activity were obtained by centrifugation. CP activity was measured according to the cleavage of a z-Arg-Arg-pNA∙2 HCl synthetic peptide substrate, which was monitored spectrophotometrically as described previously [21, 23].

### Measurement of ROS generation in HMC-1 cells

Intracellular ROS accumulation in HMC-1 cells was measured by staining cells with the green fluorescence probe H_2_DCFDA, which is rapidly oxidized to highly fluorescent DCF in the presence of intracellular H_2_O_2_, and analyzed spectrofluorometrically (model Axiovert 200). Briefly, HMC-1 cells (1 × 10^5^ cells/sample) were preloaded with 5 μM H_2_DCFDA for 30 min and washed twice with culture medium. The washed cells were incubated with or without SPs or PAF for up to 30 min at 37 °C in a CO_2_ incubator. The production of intracellular ROS was determined on a Perkin Elmer LS50B spectrofluorometer using excitation and emission wavelengths of 485 and 530 nm, respectively. All background fluorescence was subtracted using the appropriate controls.

### Statistical analysis

All reactions were performed in triplicate measurements of each experiment. Results are presented as the mean ± SEM of 3 to 6 independent experiments. One-way analysis of variance (ANOVA) was performed using the statistical software package SPSS version 20 for Windows. The post hoc comparisons of means from different groups were made by the Bonferroni post hoc test. Values were considered statistically significant when the *p*-value ≤ 0.05.

## Results

### E. *histolytica*-derived secretory products do not induce mast cell degranulation

**Figure 1.**
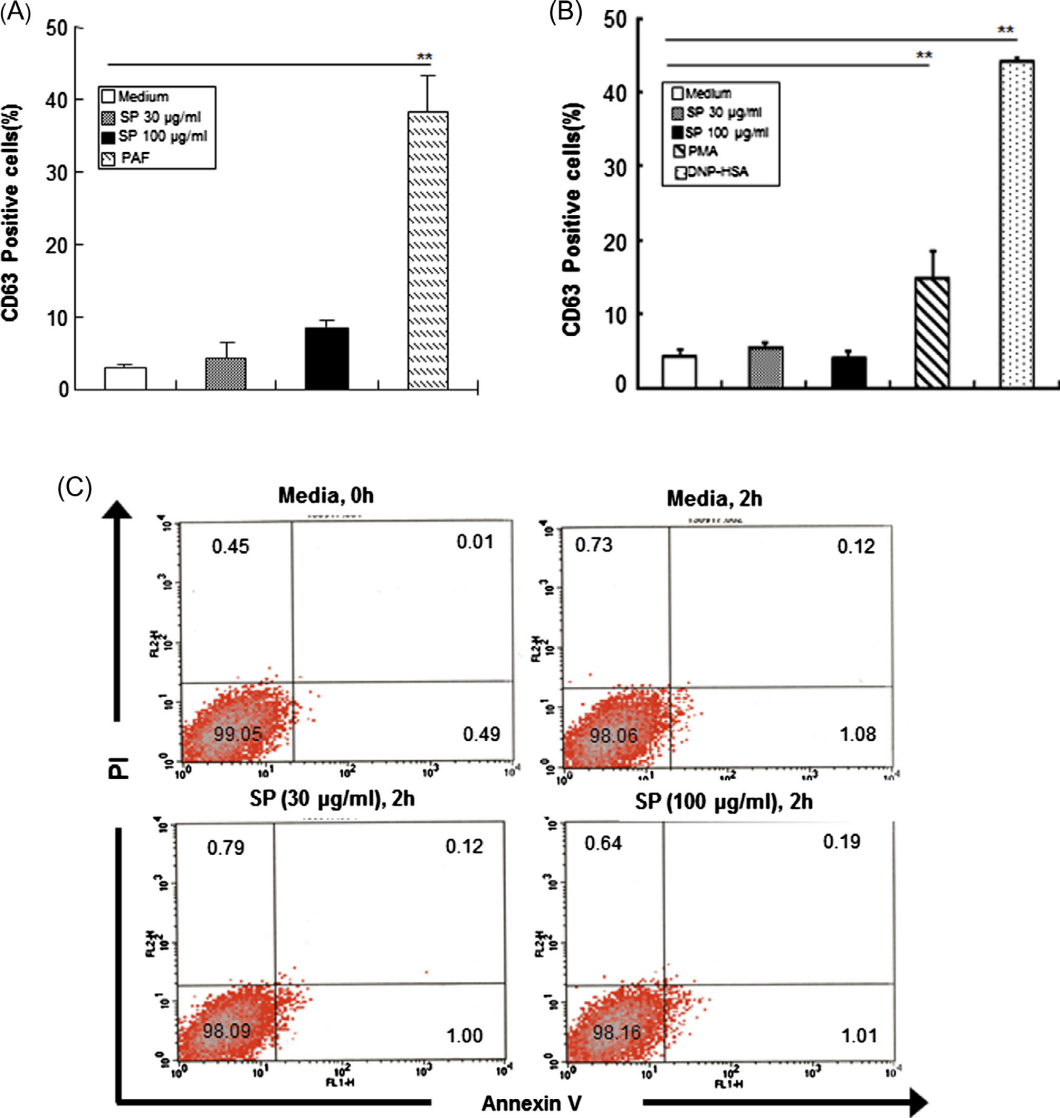
*E. histolytica*-derived secretory products (SPs) of pathogenic amoebae do not induce cell death or exocytosis in mast cells. (A) Percentage of CD63-positive cells in HMC-1 cells incubated with SPs or platelet-activating factor (PAF) (5 μM). (*n* = 3) (B) Percentage of CD63-positive cells in bone marrow-derived murine mast cells incubated with SPs, phorbol 12-myristate 13-acetate (PMA) (50nM) or DNP-HSA (250 ng/mL). (*n* = 4). (C) Flow cytometry analysis of HMC-1 cell death after stimulation with or without SPs. (*n* = 4). All reactions were performed in triplicate measurements of each experiment. All data are presented as the mean ± SEM of at least three independent experiments. The asterisks indicate the results of comparisons with the controls (**p < 0.01).

HMC-1 cells were treated with various concentrations (30 or 100 μg/mL) of SPs for 2 h to examine whether SPs could induce mast cell activation. CD63 expression is generally used as a surface marker for degranulation via exocytosis in mast cells [24]. When HMC-1 cells or BMMCs were treated with 100 μg/mL SP for 2 h, the percentage of CD63-positive cells was 8.1 ± 1.7 and 4.0 ± 0.6, respectively. However, HMC-1 cells treated with 5 μM PAF or BMMCs treated with DNP-HSA as a positive control showed a marked increase in CD63 expression (Figure 1A and 1B). At that time, there was no difference in cell viability after incubation with SPs for 2 h compared with the results for HMC-1 cells (Figure 1C) or BMMCs (data not shown) incubated with medium alone.

### Stimulation with secretory products increased IL-8 mRNA expression and protein secretion in HMC-1 cells

**Figure 2.**
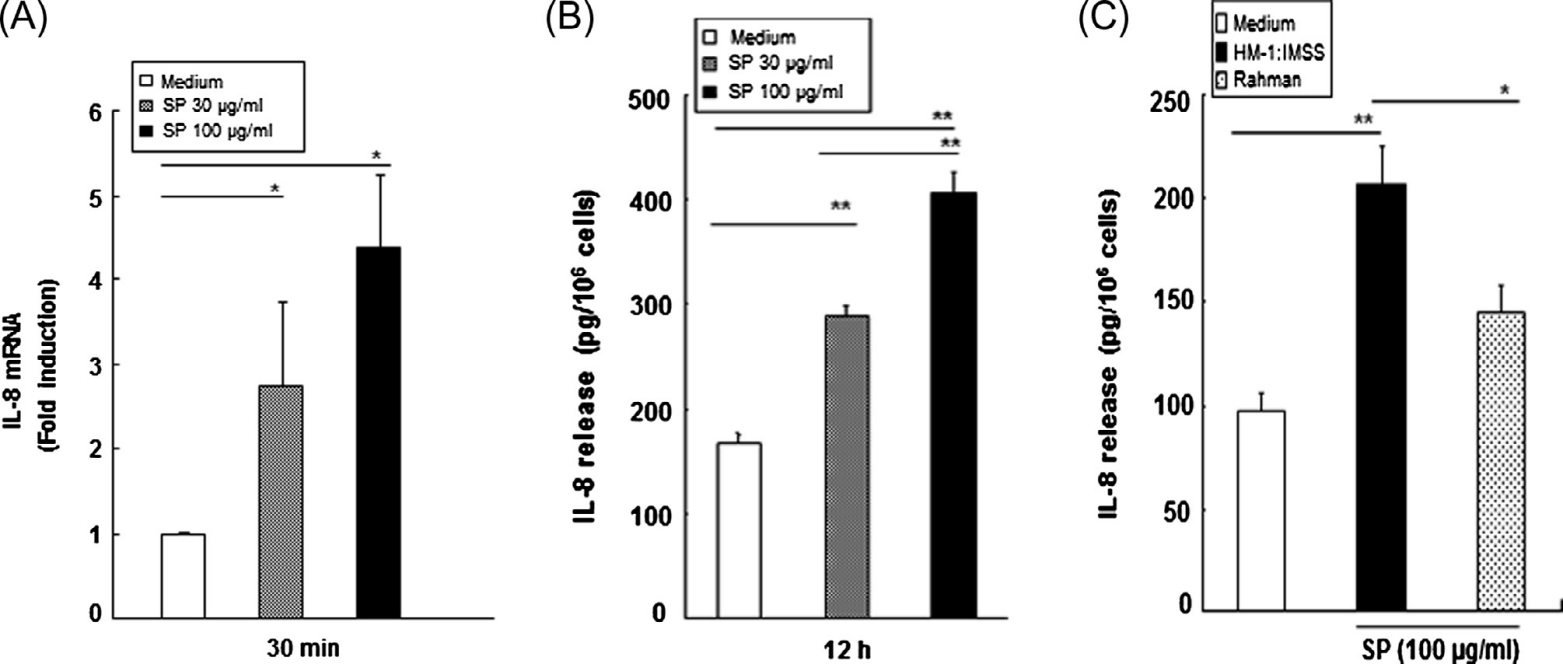
*E. histolytica*-derived secretory products (SPs) from the pathogenic wild-type HM-1:IMSS strain induce IL-8 gene expression and protein secretion in HMC-1 cells. (A) Increased *IL-8* mRNA expression in HMC-1 cells induced by SPs. (*n* = 3) (B) IL-8 production in SPs-stimulated HMC-1 cells. (*n* = 6) (C) Comparison of IL-8 production in HMC-1 cells stimulated with SPs obtained from wild-type or non-pathogenic Rahman strains. (*n* = 4). All reactions were performed in triplicate measurements of each experiment. Data are presented as the mean ± SEM of at least three independent experiments. The asterisks indicate the results of comparisons with the controls (*p < 0.05, **p < 0.01).

To examine whether SPs could induce secretion of IL-8 in HMC-1 cells, real-time PCR and ELISA analyses were performed. The real-time PCR analysis revealed that HMC-1 cells stimulated with SPs for 30 min resulted in a 3- to 4-fold increase in IL-8 mRNA as compared with cells treated with medium alone (Figure 2A). As shown in Figure 2B, SP-stimulated HMC-1 cells released IL-8 protein in a dose-dependent manner, and HMC-1 cells stimulated with SPs for 12 h resulted in a 1.7- and 2.4-fold increase in IL-8 protein at 30 and 100 μg/mL SP, respectively, compared with medium-treated cells. There was no difference in the number of annexin V-positive HMC-1 cells after incubation with SPs for 12 h compared with the results for cells incubated with medium alone (data not shown). Interestingly, SPs from the non-pathogenic Rahman strain showed a 30% reduction compared with SPs from HM-1:IMSS amoebae (Figure 2C).

### Cysteine protease activity is required for IL-8 production in HMC-1 cells induced by secretory products

**Figure 3.**
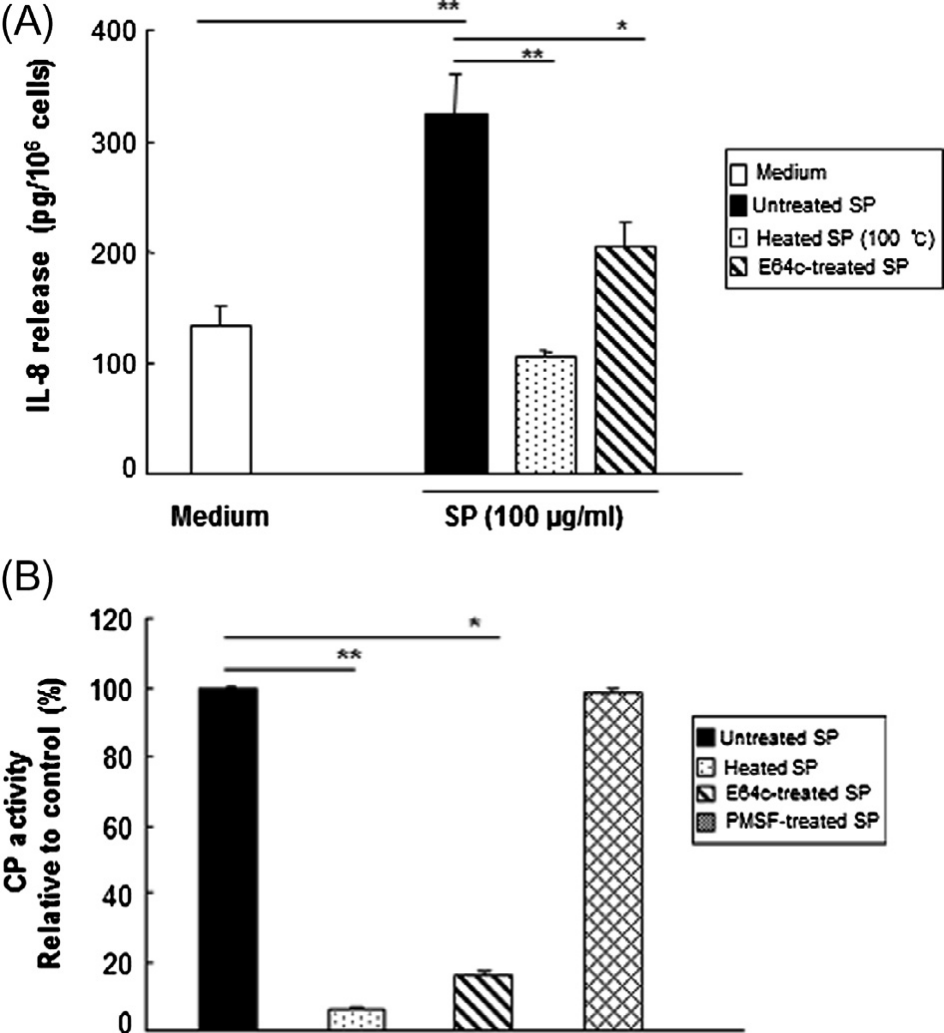
Amoebic cysteine protease activity is required for IL-8 production in HMC-1 cells induced by *E. histolytica*-derived secretory products (SPs). (A) IL-8 production in HMC-1 cells stimulated with SPs from native or modified wild-type SPs either heat-treated (100 °C, 10 min) or treated with 25 μM protease inhibitor (E64c). (*n* = 4) (B) CP activity from native or modified wild-type SPs with heat- (100 °C, 10 min) or 25 μM protease inhibitor (E64c or PMSF) treatment were measured using z-Arg-Arg-pNA·2 HCl as a substrate. The level of activity is shown as a percentage relative to the control. (*n* = 3). All reactions were performed in triplicate measurements of each experiment. Data are presented as the mean ± SEM of at least three independent experiments. The asterisks indicate the results of comparisons with the controls (*p < 0.05. **p < 0.01).

**Figure 4.**
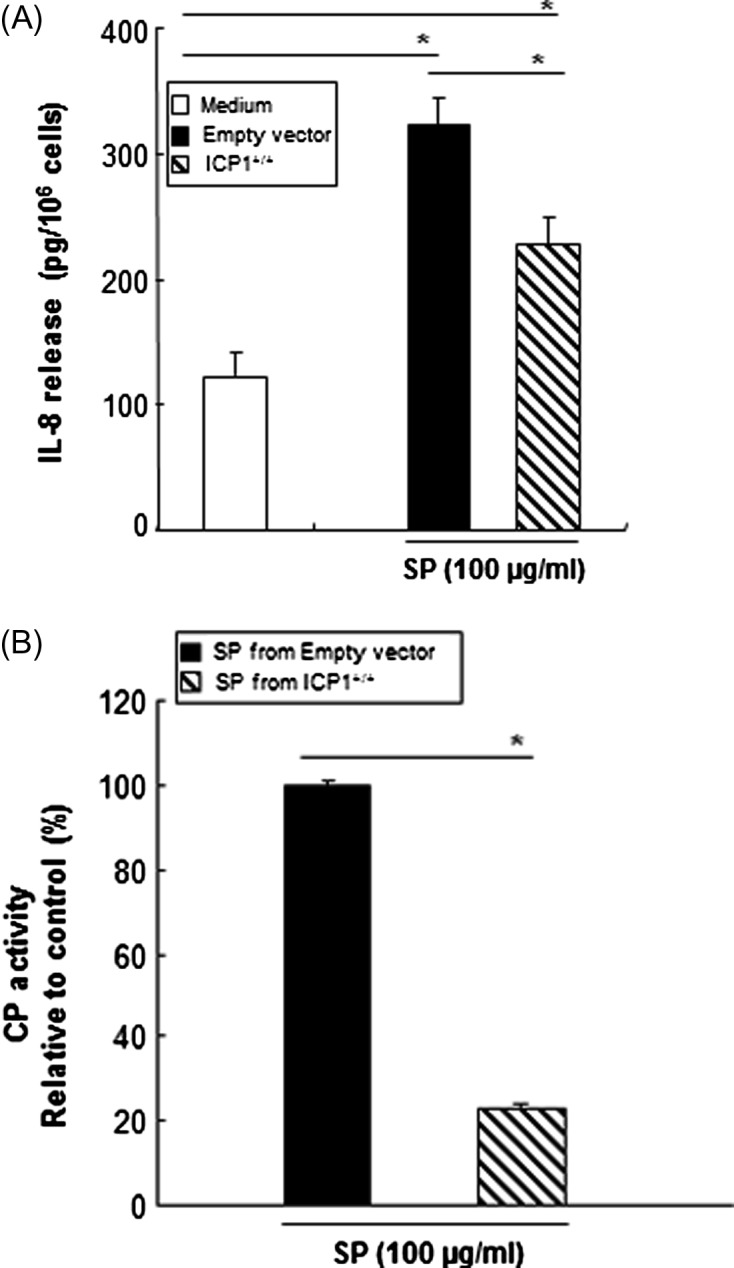
*E. histolytica*-derived secretory products (SPs) from the inhibitor of cysteine protease 1 (ICP1^+/+^) strain show a reduction in IL-8 protein secretion and cysteine protease activity in HMC-1 cells. (A) IL-8 production in HMC-1 cells stimulated with SPs from the ICP1^+/+^ or vector control strain. (*n* = 4) (B) The CP activity of SPs from the ICP1^+/+^ or vector control strain was measured using z-Arg-Arg-pNA·2 HCl as a substrate. (*n* = 3). All reactions were performed in triplicate measurements of each experiment. Data are presented as the mean ± SEM of at least three independent experiments. The asterisks indicate the results of comparisons with the controls (*p < 0.05).

To determine if amoebic CPs obtained from wild-type HM-1:IMSS SPs are responsible for SP-triggered IL-8 production in mast cells, modified SPs were incubated with HMC-1 cells. As shown in Figure 3A, the IL-8 production induced by SPs was abolished by pretreatment of the SPs with heat, suggesting that *Entamoeba*-secreted heat-labile protein components may participate in IL-8 production in HMC-1 cells. SP-induced IL-8 production in HMC-1 cells was significantly reduced by pretreatment of the SPs with the cysteine protease inhibitor E64c. Next, we compared the CP activity of native or modified SPs by treatment with specific protease inhibitors. As shown in Figure 3B, cysteine protease inhibitor E64c-treated SPs and heat-treated SPs from the wild-type significantly reduced CP activity as compared with untreated SPs. In contrast, serine protease inhibitor PMSF-treated SPs had no inhibitory effect on CP activity. To demonstrate that amoebic CP is responsible for SP-triggered IL-8 production in mast cells, we observed the IL-8 production in HMC-1 cells by SPs from the ICP1^+/+^ strain. SPs derived from the ICP1^+/+^ strain resulted in a decrease in IL-8 production compared with its transfectant control (Figure 4A). In addition, a marked reduction of CP activity was observed in SPs obtained from the ICP1^+/+^ strain compared with its vector control amoebae (Figure 4B).

### Secretory products-induced IL-8 production in HMC-1 cells occurs through a PAR2- and ROS-independent mechanism

**Figure 5.**
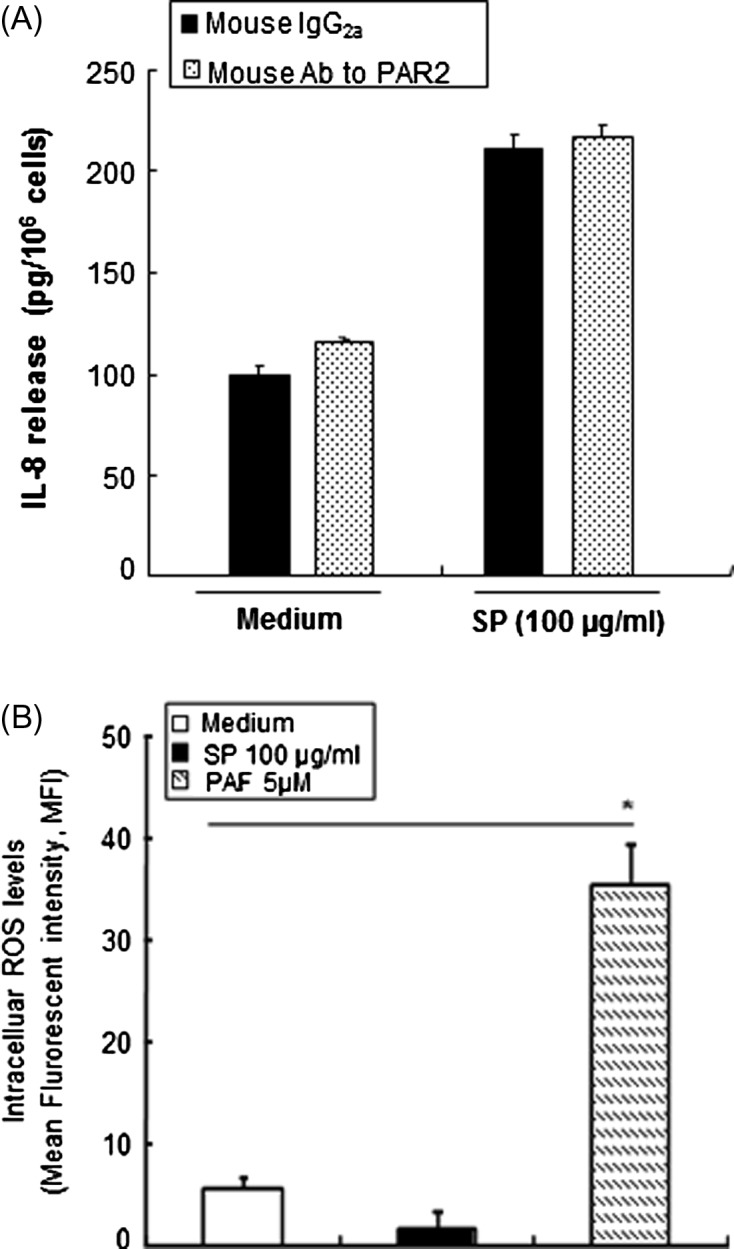
*E. histolytica*-derived secretory product (SP)-induced IL-8 production in HMC-1 cells occurs through a protease-activated receptor 2 (PAR2) or ROS-independent mechanism. (A) The effect of anti-PAR2 on SP-stimulated IL-8 production. (*n* = 4) (B) The effect of SPs on induction of ROS generation at 30 min in HMC-1 cells. (*n* = 3). All reactions were performed in triplicate measurements of each experiment. Data are presented as the mean ± SEM of at least three independent experiments. The asterisks indicate the result of comparisons with the control (*p < 0.05).

Next, we investigated whether PAR2, which is a known G-coupled receptor, is involved in IL-8 production induced by SPs. HMC-1 cells were preincubated with monoclonal Ab (10 μg/mL) to PAR2 or the isotype control IgG_2a_ for 30 min at room temperature and subsequently incubated for 12 h with or without SPs. As shown in Figure 5A, Ab to PAR2 did not inhibit SP-induced IL-8 production in HMC-1 cells. Next, we investigated whether ROS were involved in the SP-induced IL-8 production in HMC-1 cells. As shown in Figure 5B, intracellular ROS were not detected in SP-stimulated HMC-1 cells within 30 min. In contrast, intracellular ROS levels were strongly increased in HMC-1 cells stimulated with PAF as a positive control.

## Discussion

In this study, our results show that amoebic CP participates in IL-8 production through a PAR2-independent pathway in HMC-1 cells. *E. histolytica*-derived SPs containing serine or amoebic CPs have been shown to constantly secrete CPs into the extracellular environment [1, 18]. The SPs of the Rahman and ICP1^+/+^ strains resulted in a significant decrease in IL-8 production in HMC-1 cells compared with the SPs of wild-type *Entamoeba,* suggesting that a reduction in CP activity may result in a significant reduction of SP-induced IL-8 production in HMC-1 cells. Our data indicate that amoebic CP might be involved in IL-8 production in HMC-1 cells.

Recent work has shown that *E. histolytica* SPs can markedly increase IL-8 mRNA expression and protein production in colonic epithelial cells [9]. In addition, recent studies have provided evidence that chemokines such as IL-8 are crucial mediators in inflammation and in tissue injury in intestinal inflammation. IL-8 is a small, 8- to 11-kDa secreted protein that may participate in immune and inflammatory responses through chemoattraction and activation of neutrophils or leukocytes [1]. *E. histolytica* invades the intestinal mucosa and causes amoebic colitis and severe ulceration. Analysis of the inflammatory response during intestinal amoebiasis in human and animal models of the disease has revealed an important regulatory role for chemokines and cytokines. Recruitment and activation of inflammatory cells are modulated by secreted amoebic factors. SPs contain many components including cysteine protease (CP), serine protease, other proteases, phosphatases and prostaglandin E2 (PGE2).

In our preliminary experiment, we got IL-8 results of HMC-1 cells stimulated with five various concentrations (0, 10, 30, 100, and 200 μg/mL) of SP. However, no effect of 10 μg/mL of SP on IL-8 production was observed. In addition, the highest concentration of SP (200 μg/mL) showed a cytotoxic effect on HMC-1 cells (about 10% of cells were dead for 12 h), although the amount of IL-8 release in SP (200 μg/mL)-stimulated cells showed a similar level compared with the result of stimulation with 100 μg/mL (data not shown). As a result, we chose 30 and 100 μg/mL concentrations for all experiments.

In the present study, the amount of IL-8 production in HMC-1 cells induced by heat-treated SPs (100 °C for 10 min) abrogated IL-8 production, suggesting that heat-labile proteins participate in IL-8 production in HMC-1 cells. Interestingly, this result is consistent with the fact that the RGD motif in pro-mature CP5 (PCP) binds to integrin of colon cells and induces NF-kB-mediated IL-8 production in Caco-2 cells [15]. In addition, SPs from the Rahman strain resulted in a significant decrease in IL-8 production in HMC-1 cells, whereas IL-8 production by the ICP1^+/+^ strain, which is deficient in CP, was slightly diminished. This result is in agreement with the report that the Rahman strain decreased CP expression. According to a previous report [9], PGE2 participation in SP-induced IL-8 production was demonstrated in colon cells, where boiled amoebic secretory product (100 °C for 30 min) abolished SP-stimulated IL-8 production. However, involvement of the lipid mediator PGE2 in SP-stimulated IL-8 production in HMC-1 cells was not investigated in this study.

There is no information on how SPs can induce mast cell activation such as IL-8 release. G-coupled receptors or Toll-like receptors (TLR) residing on the mast cell surface may act as the biological sensor for various infectious agents during the process of mast cell activation. For example, certain proteases, including serine protease and trypsin, are signaling molecules that regulate cells by cleaving and triggering PARs [27]. Accordingly, involvement of the PAR2 receptor that is activated by serine protease and occasionally by cysteine protease was tested. In particular, PAR2 is closely related to inflammation [8]. Although SPs contain many kinds of proteases including serine, cysteine and aspartic proteases, SP-induced IL-8 production in mast cells did not occur via the PAR2 receptor. Also, it is well known that TLRs act as biological sensors of various infectious agents (i.e., viruses, bacteria, or fungi) or their products (such as lipopolysaccharide, lipoteichoic acid, and peptidoglycan) and are expressed by various innate immune cells (i.e., macrophages, neutrophils, or dendritic cells) [7, 17]. In addition to recognizing external dangers, TLRs also regulate the immune response by recognizing endogenously produced danger signals including necrotic cells, heat shock proteins, or ECM breakdown products [5]. As such, TLRs may participate in SP-induced mast cell activation; however, more experimentation is needed to investigate TLR involvement in response to SPs.

In conclusion, we have demonstrated that CPs present in SPs contribute to IL-8 production in human mast cells. Additionally, non-pathogenic Rahman and the CP-deficient ICP1^+/+^ strains showed a decrease in IL-8 production, suggesting the involvement of amoebic CP in the host cell response induced by *E. histolytica* infection, helping us to understand the mechanism of pathogenesis in *E. histolytica*.
